# Vestige: Maximum likelihood phylogenetic footprinting

**DOI:** 10.1186/1471-2105-6-130

**Published:** 2005-05-29

**Authors:** Matthew J Wakefield, Peter Maxwell, Gavin A Huttley

**Affiliations:** 1 Predictive Medicine Group, John Curtin School of Medical Research, The Australian National University, Canberra 0200 ACT Australia; 2 ARC Centre for Kangaroo Genomics, John Curtin School of Medical Research, The Australian National University, Canberra 0200 ACT Australia; 3 Computational Genomics Laboratory, John Curtin School of Medical Research, The Australian National University, Canberra 0200 ACT Australia; 4 Centre for BioInformation Science, John Curtin School of Medical Research, The Australian National University, Canberra 0200 ACT Australia

## Abstract

**Background:**

Phylogenetic footprinting is the identification of functional regions of DNA by their evolutionary conservation. This is achieved by comparing orthologous regions from multiple species and identifying the DNA regions that have diverged less than neutral DNA. Vestige is a phylogenetic footprinting package built on the PyEvolve toolkit that uses probabilistic molecular evolutionary modelling to represent aspects of sequence evolution, including the conventional divergence measure employed by other footprinting approaches. In addition to measuring the divergence, Vestige allows the expansion of the definition of a phylogenetic footprint to include variation in the distribution of any molecular evolutionary processes. This is achieved by displaying the distribution of model parameters that represent partitions of molecular evolutionary substitutions. Examination of the spatial incidence of these effects across regions of the genome can identify DNA segments that differ in the nature of the evolutionary process.

**Results:**

Vestige was applied to a reference dataset of the *SCL *locus from four species and provided clear identification of the known conserved regions in this dataset. To demonstrate the flexibility to use diverse models of molecular evolution and dissect the nature of the evolutionary process Vestige was used to footprint the Ka/Ks ratio in primate *BRCA1 *with a codon model of evolution. Two regions of putative adaptive evolution were identified illustrating the ability of Vestige to represent the spatial distribution of distinct molecular evolutionary processes.

**Conclusion:**

Vestige provides a flexible, open platform for phylogenetic footprinting. Underpinned by the PyEvolve toolkit, Vestige provides a framework for visualising the signatures of evolutionary processes across the genome of numerous organisms simultaneously. By exploiting the maximum-likelihood statistical framework, the complex interplay between mutational processes, DNA repair and selection can be evaluated both spatially (along a sequence alignment) and temporally (for each branch of the tree) providing visual indicators to the attributes and functions of DNA sequences.

## Background

Phylogenetic footprinting is a computational approach to finding functional elements in DNA by comparing sequences between species. In an evolutionary context the 'footprint' is the altered pattern of divergence resulting from a functional constraint. This is typically estimated as a reduced number of sequence changes. Phylogenetic footprinting was popularised by Tagle *et al*.[1] who demonstrated its utility in analysing the beta globin gene cluster. As larger amounts of non-coding DNA became available, easy to use tools such as Pipmaker were developed promoting wider adoption and acceptance of the technique[2,3].

Interest in phylogenetic footprinting was increased when comparison of the mouse and human genomes showed a surprisingly high proportion of the genome could be aligned and that at least 1.5% of the genome was highly conserved non-repeat, non-protein coding DNA[4].

The conventional phylogenetic footprinting method relies on the comparison of a measure of evolutionary distance to determine conservation. Since the initial development of Pipmaker several groups have sought to improve upon the simple pair-wise percent identity measure of evolutionary distance. Chapman *et al.*[5] used alignment score from a multi species alignment. Margulies *et al.*[6] define a multi-species conserved site, using a parsimony and binomial probability method to score the importance of a match in each species. The binomial approach calculates the probability of observed or greater conservation with a reference sequence in a window given a neutral substitution rate (calculated from fourfold degenerate sites in codons) for that species. Equal levels of sequence conservation are given different scores, depending on their local neutral substitution rates. For example, a higher score is awarded where there is a higher neutral substitution rate. An averaging that halves the contribution of a species to the score at each node in the tree is then applied to reduce the weighting bias effect of non-symmetrical tree topologies. The parsimony model calculates a parsimony score for each column in the alignment and assigns a P-value based on simulations of parsimony scores for data generated under the HKY85 model calibrated with a previously determined phylogenetic tree and branch lengths.

The methods of Margulies *et al*. and Chapman *et al*. both improve the sensitivity of detecting changes in divergence by including additional species in their calculation, reducing the probability of conservation due solely to chance.

Likelihood methods for analysing sequence evolution have been widely adopted in the molecular evolution community for their advantages in consistency, sufficiency and the ability to naturally compare hypotheses. By definition, parsimony is minimum evolution, and will be biased towards underestimation, especially with longer branch lengths where the probability of multiple events at the same site increases.

Central to the likelihood-based approach are continuous time Markov process models of substitution. The states in this Markov chain correspond to elements in the respective sequence alphabet, and will subsequently be referred to as motifs. The probability a motif changes (or remains the same) can be parameterised in many ways, e.g. according to biochemical attributes of the motifs involved. Motifs can be individual nucleotides or amino acids, biologically meaningful groups such as the triplets of nucleotides that make up a codon, or artificial groupings designed to capture dependencies such as dinucleotides. The Markov process for modelling motif changes is represented as a matrix of average relative rates of change and the matrix of substitution probabilities for a given time period is determined by a matrix exponentiation procedure. Details of these molecular evolution methods can be found elsewhere [7-9].

Methods using probabilistic evolutionary modelling for phylogenetic footprinting have been applied in two previous publications. Boffelli et al[10], who call their method phylogenetic shadowing, utilized fastDNAml[11] to determine mutation rates under the HKY85 model[12] for conserved and non-conserved regions from a training set of closely related primate data. These fitted models were then used for a likelihood ratio test to determine the probability of a given alignment column being in the conserved or non-conserved state and the likelihood ratios averaged over windows. The UCSC genome browser PhyloHMM track uses a combined HMM and probabilistic model of evolution to develop a HMM categoriser that can distinguish previously trained states (e.g. conserved vs. non-conserved). This model includes dependency of a nucleotide on the preceding site and implements a fully parameterised model analogous to a general time reversible model[13].

All of the previously discussed programs and strategies consider only measures of the expected number of substitutions per motif. This expected number of substitutions derives from the product of substitution rate and time, and reflects the combined influence of all mutational and selective processes. Although looking at low values of this statistic has proven an effective method for identifying regions that have a biological function that constrains their evolution, it does little to provide answers to the how and why of sequence divergence.

By examining both the degree of conservation and individual components of the evolutionary processes, it should be possible to elucidate and infer the nature of evolutionary processes occurring at different sites in the genome. Using models that include terms for biologically relevant sequence changes, and tracking the change in estimates of the model terms along the sequence, regions where specific biological processes are predominating can be identified. The models can then be tailored to both the properties of the sequence being analysed (e.g. coding, or intergenic), and the effect of the biological process of interest.

Vestige allows an examination of the temporal component of substitutions. Branches on a tree represent episodes of evolutionary time. Two regions may have experienced a similar amount of evolution when the entire tree is considered, but in very different ways. The extent to which such relative shifts in rate occur can be evaluated by examining the spatial distribution of a substitution statistic (such as length) for individual branches of a tree. Recent evidence of shifts in the evolutionary process between different mammalian lineages[14,15] suggests that in order to accurately assess the spatial distribution of evolutionary processes, changes in the temporal distribution across the tree will also need to be taken into account. The combination of both the temporal and spatial partitioning therefore provides a useful tool with which to illuminate processes that may have occurred in a restricted region and stopped millions of years ago or continue to occur in current populations.

## Implementation

Vestige is implemented in the Python scripting language[16]. This allows rapid development and reuse of components by advanced users. The package utilises the PyEvolve toolkit[17], building upon its performance optimisations and capacity for flexible construction of existing and novel models of molecular evolution.

The implementation of Vestige is as an extensible framework. Predefined scripts for footprinting (whole tree and per branch) of length, transition/transversion ratio, and Ka/Ks ratios are distributed with the package. These can be run as command line executable python scripts (unix or windows) or as GUI "droplets" under MacOSX. Command line arguments can be given to alter parameters such as the size of the analysis window and the step size the analysis window advances.

These simple control scripts can be modified to alter the model of evolution, apply constraints on model terms and specify the terms from the model that are visualized, creating a new distributable and easy to use script.

Vestige is implemented with parallelization at the per window level and will automatically use any available additional processors when run in an MPI environment.

Two key sequence manipulations must be performed prior to analysis using Vestige. As alignment algorithms are a fast moving and specialized field, it was decided not to integrate any alignment algorithm into the Vestige package but require user-supplied alignments. Vestige also does not do any automated masking of repeats. Masking of repeats sequences such as transposable elements can be done by the user prior to footprinting by Vestige using programs such as RepeatMasker[18] to generate masked sequence prior to alignment. This may be desirable as repeats may not share the same phylogeny and may show a high level of conservation that can visually clutter the analysis. Alternatively, these can be annotated and their influence on the analysis assessed visually.

Probabilistic phylogenetic modelling requires a phylogenetic tree. For reversible substitution models, an unrooted tree is used with the result that for two and three sequence cases only one unrooted tree is possible. In these cases, no phylogenetic inference is necessary and Vestige constructs the tree. In the case of more than three sequences the user can either supply a tree topology or Vestige will automatically construct a neighbour joining tree using the PyEvolve toolkit[19]. For some groups of species certain tree topologies are accepted by the community such as the Murphy *et al*.[20] mammal tree, and should be used as they represent a more robust reconstruction than is possible with a single sequence.

Visualization is an important aspect of phylogenetic footprinting packages, as analysis often involves the integration of complex data from multiple sources. The drawing of Vestige results utilises the ReportLab[21] library, outputting PDF files that can be edited in many illustration packages and are suitable for high quality publications. Due to the familiarity of many potential users of the package with existing software that displays conserved regions as a peak (high Y value) on a graph, it was decided to mimic this format, even though the natural mapping of branch lengths would be to display short evolutionary conserved sequences as zero. As evolutionary distance varies from zero to (theoretically) positive infinity, a transformation that clearly displays all values and provides good discrimination between values close to zero was required. For this reason, distances are plotted as e^-length^.

The standard statistics displayed in a Vestige run are the sum of the branch lengths in the tree for each window and the individual branch lengths for each branch and ancestral node in the tree. This allows examination of changes in the spatial pattern of divergence for each evolutionary episode, and visual assessment of the consistency of a signal between regions of the tree and the entire tree.

In addition to the commonly used distance measurement, any parameter of the model of substitution can be estimated and plotted, either globally for the entire tree or individually for each branch, providing an indication of the spatial and temporal distribution of the process that is represented by that term. Although Vestige is implemented to allow the user maximum flexibility in model specification and parameter scoping, care must be exercised to avoid overreaching the capabilities of the model or data. Users should be wary of over parameterisation, which will result in estimates with very large variance. To some extent this problem can be addressed by using larger window sizes and/or global (whole tree) rather than local (branch specific) scope for parameters. Ultimately, there is a trade off between detecting individual short sequence elements and the accuracy of estimation.

To aid in the interpretation of regions of conservation the GFF[22] and Genbank[23] annotation formats can be used to provide flexible multi-track decorations on the alignment to integrate data from multiple sources. The way that annotations are interpreted, grouped and displayed can readily be customized in user scripts. Annotation of biologically known features allows visual comparison between the level of divergence (or other parameters) of previously identified features and confirmed functional regions as a guide to importance of novel regions.

The Vestige package is conceived as a data mining and hypothesis generation tool rather than a strict hypothesis testing framework. Correcting for multiple non-independent tests from a sliding window analysis is a difficult problem. Instead, we suggest an effective solution is visual inspection that draws on functional annotations as a reference coupled with an indication of the uncertainty of parameter estimates. From this perspective, novel predictions based on phylogenetic footprinting require validation. Accordingly, Vestige does not calculate test statistics for assessing the significance of conservation. Instead, the support for parameter estimates is provided in the form of a 95% confidence interval, estimated for branch specific parameters and global parameters. This is determined by fixing the values of all other parameters at their maximum likelihood estimates and calculating the likelihood for different values for the parameter until the point at which the likelihood ratio differs by the amount equivalent to a 5% probability in a one degree of freedom chi-squared test[24]. As is the case for all parametric statistical models, the accuracy of parameter estimates increases with increasing data (bigger window sizes). Users should remain mindful of this fact when inspecting all estimates, and that by chance one window in twenty are expected to lie outside this range. Further guidance to the importance of a conservation signal is given by indicating the top 5% of windows for the summed distance measure.

The PyEvolve toolkit provides global and local optimisers for estimating model parameters. The global simulated annealing optimiser is capable of finding the global optima in complex functions with multiple optima, while the fast Powell optimiser is more prone to falling into local optima. To improve the speed of Vestige analysis, a global estimate of parameters is generated by simulated annealing optimisation of either the entire alignment or a random sample of columns drawn without replacement. This estimate is then used as a starting point by the Powell optimiser for rapid analysis or the more robust simulated annealing optimiser. To identify any windows that may suffer from poor optimisation a graph of the absolute value of the log-likelihood is included on the output. Abrupt discontinuities in the graph indicate optimisation problems. The user can then re-run the analysis with more robust optimisation settings.

Vestige determines the frequency of the sequence motifs from the alignment. By default, the frequency of motifs in the entire alignment is used or the user can select to use the frequency in only the window being analysed.

The ability to employ models of substitution with motif sizes greater than 1 raises the issues of generating invalid motifs, and inaccurate estimation of motif probabilities.

Invalid motifs can be generated when the window is advanced into a different frame, and motifs are observed that were not in the original frame. For example, if the codon sequence (ATG) (AAG) is analysed in the second frame the (TGA) stop codon motif occurs, an invalid state for codon models of substitution[25]. Vestige addresses this problem using a flag that asserts that step should only be a multiple of the motif size.

Inaccurate estimation of motif frequencies can occur when using models with motif sizes greater than one, as motif frequencies are normally counted only in the current frame. If a motif is rare in the frame used to calculate the motif frequency, but occurs frequently in the current analysis frame, the likelihood for windows in the current frame will be significantly decreased. Vestige therefore calculates an average motif frequency across all frames when using global motif probabilities for models with motif sizes greater than one.

## Results and discussion

To demonstrate the functionality and broad utility of the Vestige package we present two analyses, the *SCL *locus analysed with a dinucleotide model, and exon 11 of 5 primate *BRCA1 *sequences "footprinted" for the ratio of Ka/Ks model terms using a codon model.

The alignment of human, mouse, rat and dog totalling 128 kb from the *SCL *locus and its annotations in GFF format are those used by Chapman *et al *allowing direct comparison with their results[5]. The *BRCA1 *alignment is identical to that used by Huttley[26] with redundant gaps removed.

**Figure 1:**
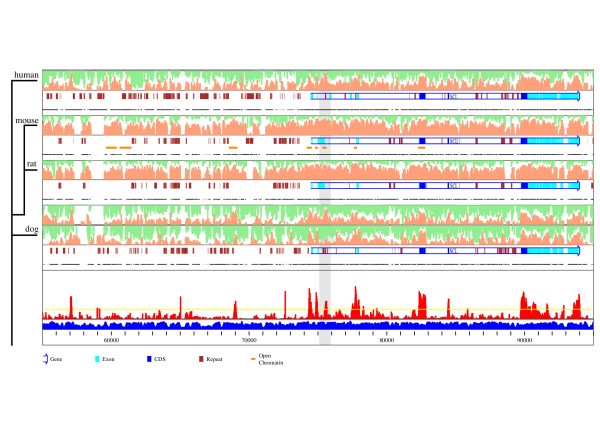
**Footprinting the SCL gene**. Phylogenetic footprinting of the genomic region around the SCL gene. The alignment of Chapman et al.[5], with their experimentally determined regions of biological importance annotated, was footprinted in 100 bp windows with a 25 bp step using a dinucleotide model of evolution based on the HKY85 model[12]. This model contains terms for the frequency of each dinucleotide (taken from the complete 139 kb alignment) and for the transition/transversion ratio which is applied when the difference between the dinucleotides is a transition. The total branch length summed across the tree is plotted as e^-length ^in red and the absolute value of the log likelihood (smaller is better) in blue. The yellow line indicates the level of conservation of the top 5% of windows for the entire 139 kb alignment. Local branch lengths are presented in 5 panels aligned with a stepped dendrogram representation of the phylogenetic tree. Annotations for each species are displayed below the graph, with the lower black lines representing sequence and white space gaps. Coloured annotations in the upper track are described below the mainplot. The fourth track is the derived ancestor of mouse and rat, and therefore has no sequence or annotation. The display of local branch lengths consists of a plot of the length at the lower bound of the 95% confidence estimate in salmon, and the upper bound of the 95% confidence estimate in green. The 95% confidence interval estimate for the branch length is represented by the white space between these graphs. Regions of high confidence conservation can be identified by looking for peaks in the lower salmon graph, and conversely regions of high confidence divergence can be identified by identifying hanging peaks in the upper green graph. Regions where no reliable branch length estimate can be given will appear white. Individual branch lengths can be compared to changes in annotation between branches. For example, the grey boxed region highlights a high confidence signal of divergence in dog between 75000 and 75300. This region correlates with part of an exon and a region of open chromatin in mouse, but is intronic in dog. This suggests that the open chromatin region will be altered or not present in dog, potentially altering regulation and function of this gene. Full analysis of the entire 139 kb region around the SCL gene is provided as a supplementary file.

The results for globally summed branch lengths across the *SCL *locus provided similar results to those obtained by Chapman *et al*. All of the experimentally determined and biologically important conserved regions in this sample alignment were detected (figure 1). Like other phylogenetic footprinting methods, Vestige fails to find any conservation at the region 8–9 kb upstream of the *SCL *start site. This site represents an altered chromatin structure in mouse and may be due to a non-DNA sequence specific change in chromatin.

Graphing statistics for individual branches on the tree provides a mechanism for discriminating between artefactual and biological causes of spatial heterogeneity. Artefacts can arise due to properties of the data and properties of the statistical models and numerical algorithms. Missing data are addressed by calculating the likelihood over all possible states and gaps are conventionally treated as missing data in the likelihood framework. Both missing data in sequences and gaps in multiple alignments have a structure to their occurrence that impacts on parameter estimates: gaps occur in trees in a taxonomically structured way reflecting their evolutionary origin; and, both gap and missing data symbols tend to occur in patches. When the patch size is greater or equal to the window size, the extreme case, one or more lineages without any information result and the true total tree branch length is equivalent to that of a smaller number of taxa. Yet because the optimiser still attempts to estimate values for these parameters, optimiser behaviour can introduce a systematic error – such as zero branch length creating a region of conservation – whose pattern depends on the optimiser chosen. The introduction of a gap term into the model doesn't necessarily solve this issue as they tend to be rare and insertions reversing gaps are highly improbable, causing short branch lengths. As a solution to this problem, Vestige estimates and displays a 95% confidence interval for individual branch length estimates. The minimum and maximum of the 95% confidence interval are then displayed rather than the optimised value. Windows where a large proportion of N's creates a broad (or in the extreme case infinite) confidence interval will be displayed as white space. The user can look along the graph to identify either well supported regions of conservation (the lower graph) or well supported regions of divergence (the upper hanging graph). While this capacity for establishing artefacts as the basis for a spatial distribution is essential, more interesting is the situation in which there are lineage specific effects that are biological in origin.

Biological origins for spatial heterogeneity can, in principal, originate from spatial fluctuations in a biological process that is common to all lineages or unique to a subset of lineages. To date there has been little consideration of the latter case but several studies demonstrate this is a real topic of interest. Direct evidence of differences in DNA repair between rodents and humans[27], and indirect evidence between rat and mouse[14] and between eutherian and non-eutherian mammals[15] establish the existence of differences in DNA mutation and repair between lineages. Furthermore, the existence of substitution rate heterogeneity across genomes[28,29] illustrates the existence of spatially localised differences in mutation and repair, local effects that can change character over evolutionary time. Consequently, inference regarding the property of a single region based on a summary statistic calculated for the whole tree benefits from the ability to establish that the fundamental pattern is consistent across the majority of branches on the tree. Of course, inconsistency across the tree can also be of interest since they may indicate changes in local mutagenic environment providing insight into shifts in the boundaries of biological features. An example of one such change is given in figure 1, where a region that is exonic in mouse and rat is intronic in dog. This region is conserved in all lineages except that leading to the dog. The tight confidence intervals on these estimates for this region across all lineages, coupled with the strength of signal on the lineage leading to the common ancestor of mouse and rat, suggests loss of this exon occurred on the lineage leading to dog. These observations suggest the hypothesis that the open chromatin region observed in mouse will have been lost or significantly altered in dog. Inconsistency of the spatial pattern between branches or clades can therefore facilitate identifying key regions of biological differentiation.

We have broadened the definition of a footprint to include spatial changes in estimates of any model term. These terms may be either global in scope or locally estimated for each branch. To demonstrate the utility of this broader definition we have footprinted primate *BRCA1 *for signals of adaptive evolution. This was chosen as an example as it demonstrates the ability to work with different sequence data types and with biologically meaningful model terms other than branch length. Implementation of non-standard annotations requires some scripting and the python script for this example provides a template for other applications that may require fine control over the behaviour of Vestige (script included in distribution).

**Figure 2:**
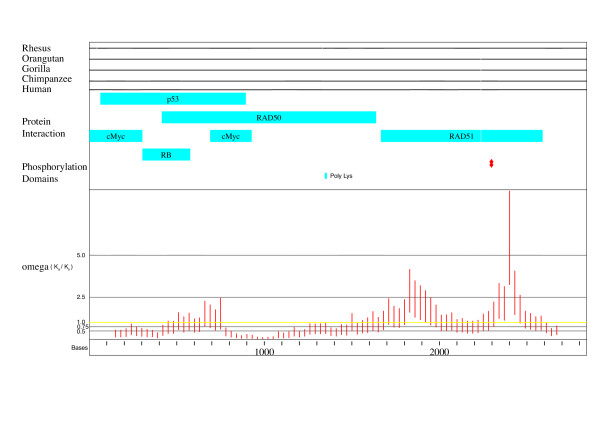
**Footprinting the Ka/Ks ratio in primate BRCA1**. A DNA alignment of five partial primate sequences from exon 11 of BRCA1 footprinted for adaptive evolution. A codon model of evolution with a model parameter for replacement changes[25] was "footprinted" in 300 base (100 codon) windows and 30 base steps. Red lines indicate the 95% confidence interval for the omega replacement parameter that is an estimate of the Ka/Ks ratio. Ka/Ks > 1 is indicative of adaptive evolution. Two regions (around 1850 and 2400 bp) have 95% confidence intervals for omega that do not include 1, suggesting adaptive evolution is occurring within them. Note that although plotted as single lines in the middle of the window range the 300 bp windows overlap and a single region or site can affect the parameter estimate for multiple adjacent windows. Annotations of protein-protein interaction domains (blue boxes) and phosphorylation sites (red diamonds) are derived from Deng[33]. Sequences: Human 961-3798 of NM007294, Chimpanzee 150-2987 of AF019075, Gorilla 150-2987 of AF019076, Orangutan 150-2987 of AF019077 and Rhesus 150-2984 of AF019078. Scale is in bases and refers to gapped alignment positions.

The Ka/Ks statistic is the ratio of non-synonymous to synonymous substitution rates, and represents the impact of natural selection. A Ka/Ks ratio of less than one indicates purifying selection, a ratio equal to one indicates selective neutrality, and a ratio greater than one is evidence of positive or adaptive evolution. The Ka/Ks ratio is modelled by adding a term (omega) to the standard codon model that applies when an instantaneous substitution results in a change in the amino acid coded for by the codon[30]. Due to the requirement of a moderate sized data set for omega estimation a 100 codon (300 bp) window and global (whole tree) scoping of the omega parameter were used. The analysis of *BRCA1 *indicates there are three regions that have Ka/Ks ratios greater than one, putatively indicative of adaptive evolution (figure 2). Two of these three regions have estimated 95% confidence intervals that do not contain the value 1, suggesting the hypothesis that adaptive modifications to BRCA1 function have occurred within them. Both regions fall within the *RAD51 *binding domain and suggest that modulation of *RAD51 *binding may be the driving force for *BRCA1 *adaptive evolution in primates. This result is consistent with the inference of Huttley *et al.*[31] and variants in *RAD51 *that modify breast cancer risk in *BRCA1 *mutation carriers[32].

## Conclusion

Vestige provides a flexible, open platform for phylogenetic footprinting that expands the range of model terms and hence biological processes that can be evaluated. The framework facilitates visualisation of results from the increasingly rich probabilistic models of molecular evolution aimed at detecting and categorizing regions of the genome.

## Availability and requirements

**Project name: **Vestige

**Project home page: **

**Operating system(s): **Platform independent

**Programming language: **Python

**Other requirements: **Python 2.3 or higher, PyEvolve 0.89 or higher, Reportlab, and Numeric Python.

**License: **GNU GPL

## Authors' contributions

MJW designed and implemented the software. PM wrote the drawing code, refactored the code during development and contributed to software and algorithmic design. GAH wrote a prototype (sans visualisation) that stimulated the current project, and provided guidance in the application of the underlying molecular evolutionary modelling methods and PyEvolve toolkit. All authors read and approved the final manuscript.

## Additional data files

• File Name: Vestige.tar.gz

• File Format: gzip compressed tar archive

• Description: The python script files, documentation and data for the software described in this paper.

• File Name: scl_vestige_dinucleotide.pdf

• File Format: Adobe Portable Document Format

• Description: The complete output of vestige analysis of the scl region, a subsection of which is presented in figure 1. See figure 1 for details.

• File Name: Vestige_MacOX_Droplets.tar.gz

• File Format: gzip compressed tar archive

• Description: Binary distribution of standalone MacOSX graphic user interface droplets, that can be used for running vestige by dragging an alignment and optionally a tree and gff file onto the application icon in the MacOSX finder. Requires MacOSX10.3

• File Name: Cogent0_89.tar.gz

• File Format: gzip compressed tar archive

• Description: Source code distribution of the PyEvolve package

## References

[B1] Tagle DA, Koop BF, Goodman M, Slightom JL, Hess DL, Jones RT (1988). Embryonic epsilon and gamma globin genes of a prosimian primate (Galago crassicaudatus). Nucleotide and amino acid sequences, developmental regulation and phylogenetic footprints. J Mol Biol.

[B2] Schwartz S, Zhang Z, Frazer KA, Smit A, Riemer C, Bouck J, Gibbs R, Hardison R, Miller W (2000). PipMaker – a web server for aligning two genomic DNA sequences. Genome Res.

[B3] Hardison RC (2000). Conserved noncoding sequences are reliable guides to regulatory elements. Trends Genet.

[B4] Waterston RH, Lindblad-Toh K, Birney E, Rogers J, Abril JF, Agarwal P, Agarwala R, Ainscough R, Alexandersson M, An P (2002). Initial sequencing and comparative analysis of the mouse genome. Nature.

[B5] Chapman MA, Donaldson IJ, Gilbert J, Grafham D, Rogers J, Green AR, Gottgens B (2004). Analysis of multiple genomic sequence alignments: a web resource, online tools, and lessons learned from analysis of mammalian SCL loci. Genome Res.

[B6] Margulies EH, Blanchette M, Haussler D, Green ED (2003). Identification and characterization of multi-species conserved sequences. Genome Res.

[B7] Felsenstein J (1981). Evolutionary trees from DNA sequences: a maximum likelihood approach. J Mol Evol.

[B8] Felsenstein J (2004). Inferring phylogenies.

[B9] Lio P, Goldman N (1998). Models of molecular evolution and phylogeny. Genome Res.

[B10] Boffelli D, McAuliffe J, Ovcharenko D, Lewis KD, Ovcharenko I, Pachter L, Rubin EM (2003). Phylogenetic shadowing of primate sequences to find functional regions of the human genome. Science.

[B11] Olsen GJ, Matsuda H, Hagstrom R, Overbeek R (1994). fastDNAmL: a tool for construction of phylogenetic trees of DNA sequences using maximum likelihood. Comput Appl Biosci.

[B12] Hasegawa M, Kishino H, Yano T (1985). Dating of the human-ape splitting by a molecular clock of mitochondrial DNA. J Mol Evol.

[B13] Siepel A, Haussler D (2004). Combining phylogenetic and hidden Markov models in biosequence analysis. J Comput Biol.

[B14] Cooper GM, Brudno M, Stone EA, Dubchak I, Batzoglou S, Sidow A (2004). Characterization of evolutionary rates and constraints in three Mammalian genomes. Genome Res.

[B15] Margulies EH, Maduro VV, Thomas PJ, Tomkins JP, Amemiya CT, Luo M, Green ED (2005). Comparative sequencing provides insights about the structure and conservation of marsupial and monotreme genomes. Proc Natl Acad Sci U S A.

[B16] Python Scripting Language. http://www.python.org.

[B17] Butterfield A, Vedagiri V, Lang E, Lawrence C, Wakefield MJ, Isaev A, Huttley GA (2004). PyEvolve: a toolkit for statistical modelling of molecular evolution. BMC Bioinformatics.

[B18] RepeatMasker. http://repeatmasker.genome.washington.edu.

[B19] PyEvolve 0.89. http://cbis.anu.edu.au/software/.

[B20] Murphy WJ, Eizirik E, O'Brien SJ, Madsen O, Scally M, Douady CJ, Teeling E, Ryder OA, Stanhope MJ, de Jong WW (2001). Resolution of the early placental mammal radiation using Bayesian phylogenetics. Science.

[B21] ReportLab Graphics Library. http://www.reportlab.org/.

[B22] GFF: an Exchange Format for Feature Description. http://www.sanger.ac.uk/Software/formats/GFF/.

[B23] The DDBJ/EMBL/GenBank Feature Table. http://www.ncbi.nlm.nih.gov/projects/collab/FT/index.html.

[B24] Burnham KP, Anderson DR (2002). Model selection and multimodel inference : a practical information-theoretic approach.

[B25] Goldman N, Yang Z (1994). A codon-based model of nucleotide substitution for protein-coding DNA sequences. Mol Biol Evol.

[B26] Huttley GA (2004). Modeling the Impact of DNA Methylation on the Evolution of BRCA1 in Mammals. Mol Biol Evol.

[B27] Bohr VA, Smith CA, Okumoto DS, Hanawalt PC (1985). DNA repair in an active gene: removal of pyrimidine dimers from the DHFR gene of CHO cells is much more efficient than in the genome overall. Cell.

[B28] Wolfe KH, Sharp PM, Li WH (1989). Mutation rates differ among regions of the mammalian genome. Nature.

[B29] Malcom CM, Wyckoff GJ, Lahn BT (2003). Genic mutation rates in mammals: local similarity, chromosomal heterogeneity, and X-versus-autosome disparity. Mol Biol Evol.

[B30] Yang Z (1998). Likelihood ratio tests for detecting positive selection and application to primate lysozyme evolution. Mol Biol Evol.

[B31] Huttley GA, Easteal S, Southey MC, Tesoriero A, Giles GG, McCredie MR, Hopper JL, Venter DJ (2000). Adaptive evolution of the tumour suppressor BRCA1 in humans and chimpanzees. Australian Breast Cancer Family Study. Nat Genet.

[B32] Jakubowska A, Narod SA, Goldgar DE, Mierzejewski M, Masojc B, Nej K, Huzarska J, Byrski T, Gorski B, Lubinski J (2003). Breast cancer risk reduction associated with the RAD51 polymorphism among carriers of the BRCA1 5382insC mutation in Poland. Cancer Epidemiol Biomarkers Prev.

[B33] Deng CX, Brodie SG (2000). Roles of BRCA1 and its interacting proteins. Bioessays.

